# Molecular Markers for Detecting *Schistosoma* Species by Loop-Mediated Isothermal Amplification

**DOI:** 10.1155/2020/8042705

**Published:** 2020-07-22

**Authors:** Pedro Fernández-Soto, Catalina Avendaño, Anna Sala-Vizcaíno, Beatriz Crego-Vicente, Begoña Febrer-Sendra, Juan García-Bernalt Diego, Ana Oleaga, Julio López-Abán, Belén Vicente, Manuel A. Patarroyo, Antonio Muro

**Affiliations:** ^1^Infectious and Tropical Diseases Research Group (e-INTRO), Biomedical Research Institute of Salamanca, Research Centre for Tropical Diseases at the University of Salamanca (IBSAL-CIETUS), Faculty of Pharmacy, University of Salamanca, 37007 Salamanca, Spain; ^2^Animal Science Faculty, Universidad de Ciencias Aplicadas y Ambientales (U.D.C.A), 111166 Bogotá, Colombia; ^3^Parasitología Animal, Instituto de Recursos Naturales y Agrobiología de Salamanca (IRNASA, CSIC), Cordel de Merinas, 40-52, 37008 Salamanca, Spain; ^4^Fundación Instituto de Inmunología de Colombia (FIDIC), 111321, , Bogotá, Colombia; ^5^School of Medicine and Health Sciences, Universidad del Rosario, 112111 Bogotá, Colombia

## Abstract

Schistosomiasis is considered a neglected parasitic disease. Around 280,000 people die from it annually, and more than 779 million people are at risk of getting infected. The schistosome species which infect human beings are *Schistosoma mansoni*, *Schistosoma haematobium*, *Schistosoma intercalatum*, *Schistosoma japonicum*, *Schistosoma guineensis*, and *Schistosoma mekongi*. This disease is also of veterinary significance; the most important species being *Schistosoma bovis* since it causes the disease in around 160 million livestock in Africa and Asia. This work was aimed at designing and developing a genus-specific loop-mediated isothermal amplification (LAMP) method for detecting the most important schistosome species affecting humans and for the species-specific detection of *S. bovis*. Bioinformatics tools were used for primer design, and the LAMP method was standardised for detecting the ITS-1 region from *S. intercalatum*, *S. haematobium*, *S. mansoni*, *S. japonicum*, and *S. bovis* DNA (generic test) and the NADH 1 gene for specifically detecting *S. bovis* (at different DNA concentrations). Detection limits achieved were 1 pg DNA for *S. mansoni*, 0.1 pg for *S. haematobium*, 1 pg for *S. intercalatum*, and 10 pg for *S. bovis*. No amplification for *S. japonicum* DNA was obtained. The LAMP designed for the amplification of *S. bovis* NADH-1 worked specifically for this species, and no other DNA from other schistosome species included in the study was amplified. Two highly sensitive LAMP methods for detecting different *Schistosoma* species important for human and veterinary health were standardised. These methods could be very useful for the diagnosis and surveillance of schistosome infections.

## 1. Introduction

Schistosomiasis is a parasitic disease caused by several species of trematode worms of the genus *Schistosoma*. It is one of the 20 tropical diseases on the World Health Organization's (WHO) list of Neglected Tropical Diseases (NTDs) [1]. The disease affects at least 240 million people worldwide and more than 779 million are at risk of contracting it [2]. The infection is endemic in 78 countries, mainly in tropical and subtropical areas, although it predominates in Sub-Saharan Africa where more than 80% of the cases occur, leading to around 280,000 deaths annually. The Global Burden of Disease study attributed 1.43 million disability-adjusted life years (DALYs) to it in 2017 [2–5].

Of the 23 *Schistosoma* species described to date, *S. mansoni*, *S. haematobium*, and *S. japonicum* are the main human species [6, 7]. Nevertheless, schistosomes also represent a health problem for animals, including ruminants, rodents, and primates. The species causing animal schistosomiasis are mainly *Schistosoma bovis*, *S. japonicum*, *S. mekongi*, *S. mattheei*, *S. curassoni*, *S. margrebowiei*, *S. leiperi*, *S. indicum*, and *S. spindale*. *S. bovis* is one of the most important ones parasitizing cattle and causing significant economic losses, affecting around 160 million animals in Africa and Asia [8, 9].

Schistosomes have a complex life cycle requiring an aquatic snail as intermediate host and a vertebrate as definitive host [10]. Schistosomiasis is acquired by direct contact with fresh water contaminated by parasite larvae (called cercariae), which have been emitted into an aquatic environment by the aquatic snails, actively penetrating the skin of a susceptible host [11]. Paired couples of adult schistosome worms live in a definitive host's mesenteric or perivascular veins where they reproduce and lay their eggs. The eggs are released into the environment through urine (*S. haematobium*) or faeces (the rest of the species) or can be retained in host tissues where they induce an inflammatory response [7].

Both *S. mansoni* and *S. haematobium* are found in Africa and the Middle East, whereas *S. mansoni* is the only species found in South America. *S. japonicum* occurs in Asia, especially in the Philippines and China; *S. mekongi* in the Mekong river basin, and *S. guineensis* and *S. intercalatum* in West and Central Africa [7]. *S. bovis* can be found throughout the African continent, south-western Asia (Israel, Iran, Iraq, Syria, and Turkey), Mediterranean islands (Corsica, Sardinia, and Sicily), and the Iberian peninsula [12].

Schistosomiasis can be treated if an accurate diagnosis is made and a prompt treatment with praziquantel (PZQ) is administered. Using appropriate and sensitive diagnostic techniques is thus essential for identifying infected individuals [13]. Parasitological diagnosis is specific, cheap, and simply performed. However, in laboratories with limited resources, it is not very sensitive, especially when infection intensity is low, as occurs in areas with low prevalence and/or in individuals having been recently infected or having low parasite load. Furthermore, this can only be done after egg production and elimination has begun, approximately two months after infection [11]. Immunodiagnostic tests have been shown to have high sensitivity in cases where parasitological techniques have provided false negative results [13]. However, they have problems related to obtaining antigens and false positive results since it is difficult to differentiate between active and/or past infections or reinfections and there can also be problems regarding specificity with other helminths or even between different species from the genus *Schistosoma*. Furthermore, such tests are not useful during the disease's acute phase, since antibodies targeting the parasite would not yet have appeared [11, 13]. On the other hand, immunodiagnostic tests based on detecting the circulating cathodic antigen (CCA) or circulating anodic antigen (CAA) in either urine or blood have the advantage of not requiring a trained personnel for their interpretation or specialised equipment, being more sensitive and specific than egg detection in faeces by microscopy, although they could give false positive results. It is worth highlighting that such techniques detect adult forms and not eggs, so xenomonitoring combining either CAA or CCA with molecular biology techniques is thus recommended for verification and maintaining elimination [14].

Molecular diagnosis is particularly useful regarding infections with low parasitaemia [15, 16]. PCR and its variants have been of great use, and some authors have proposed such techniques as the gold standard for diagnosing schistosomiasis [17]. However, they are expensive and require a specialised personnel and equipment, meaning that they are not useful for diagnosis in field conditions and their use is limited to just a few reference laboratories [18].

Several molecular techniques based on isothermal methods exist, such us nucleic acid sequence-based amplification (NASBA, also known as transcription-mediated amplification, TMA), signal-mediated amplification of ribonucleic acid (RNA) technology (SMART), helicase-dependent amplification (HDA), recombinase polymerase amplification (RPA), rolling circle amplification (RCA), multiple displacement amplification (MDA), loop-mediated isothermal amplification (LAMP), and strand displacement amplification (SDA); such techniques might provide an alternative tool regarding other more complex molecular methods [19]. The development and application of new methods meeting the characteristics for the ideal diagnosis of schistosomiasis should include high sensitivity and specificity, ease of use and interpretation, being able to use different sample types, rapidity, low cost, and being able to be applied in disease-endemic areas having scarce economic resources [20]. This work describes designing and developing a LAMP method for detecting species-specific *S. bovis* and a genus-specific LAMP method for detecting the most important schistosome species affecting humans.

## 2. Materials and Methods

### 2.1. Selecting Targets for LAMP Amplification of *Schistosoma bovis* and Genus *Schistosoma*

When this study started, the *S. bovis* genome had not been yet completely sequenced and there was limited sequence information in databases. Thus, a thorough search in the GenBank database (https://www.ncbi.nlm.nih.gov/genbank/) was carried out to locate all possible available DNA sequences. An alignment of the sequences found was carried out using ClustalW to obtain a consensus sequence. When the comparison did not allow generating a consensus sequence, different sequence groups were made up based on their greater identity. Subsequently, the BLAST program (Basic Local Alignment Search Tool; https://blast.ncbi.nlm.nih.gov/Blast.cgi) was used to assess the identity of *S. bovis* sequences obtained to other species. Then, to refine the search and obtain greater accuracy in the results, the sequences were compared in two other schistosome-specific databases: SchistoDB (Schistosoma Genomic Resources; http://schistodb.net/schisto/), which contains the genome of *S. mansoni*, *S. haematobium*, and *S. japonicum*, and the Wellcome Trust Sanger Institute database (http://www.sanger.ac.uk/), which houses continuously updated genome sequencing results of 50 helminths, including several *Schistosoma* species (50 Helminth Genomes Project; http://www.sanger.ac.uk/science/collaboration/50hgp). Once all *S. bovis* sequences were compared and analysed, the most suitable one was selected for designing specific primers for the LAMP.

Useful sequences for designing the specific primers for developing a LAMP method for amplifying the genus *Schistosoma* were selected following similar steps as those described above for *S. bovis.*

### 2.2. Designing LAMP Primers

LAMP primer sets complementary to the selected specific nucleotide sequences were designed using both the online PrimerExplorer V5 software (Eiken Chemical Co., Ltd., Japan; https://primerexplorer.jp/e/) and the LAMP Designer software (OptiGene Ltd., UK; http://www.optigene.co.uk/lamp-designer/) since the two programs use different design parameters. HPLC grade primers were used (Thermo Fisher Scientific Inc., Madrid, Spain). Lyophilised primers were resuspended in ultrapure water to a final 100 pmol/*μ*L concentration and stored at -20°C until use.

### 2.3. Obtaining and Preparing *Schistosoma* Species DNA

*S. bovis* adult worms were obtained from hamsters experimentally infected in the laboratory of Animal Parasitology, Institute of Natural Resources and Agrobiology of Salamanca (IRNASA-CSIC), Spain. *S. bovis* genomic DNA (gDNA) was extracted from worms kept frozen using the NucleoSpin Tissue Kit (Macherey-Nagel, GmbH&Co., Germany) following the manufacturers' instructions.

*S*. *mansoni* DNA (Brazilian strain) was extracted from frozen adult male and female worms available in our laboratory. This strain has been maintained by serial passages in mice routinely infected in the Laboratory of Parasitic and Molecular Immunology, CIETUS, University of Salamanca. Genomic DNA from adult male and female *S. haematobium* (Egyptian Strain; NR-31682) and genomic DNA from adult male and female *S. japonicum* (Chinese Strain; NR-36066) were obtained from the Schistosomiasis Resource Centers for distribution by BEI Resources, NIAID, NIH (https://www.beiresources.org/Collection/51/Schistosome-Resource-Centers.aspx). *S. intercalatum* DNA was provided by Doctor José Manuel da Costa from the Center for Parasite Biology and Immunology, National Institute of Health Doutor Ricardo Jorge, Porto, Portugal. This DNA comes from a donation from Centers for Disease Control and Prevention, Atlanta, USA. All gDNAs were measured three times by spectrophotometry using a Nanodrop ND-100 spectrophotometer (Nanodrop Technologies) to obtain an average concentration and then diluted with ultrapure water to a final 5 ng/*μ*L concentration. Subsequently, serial 10-fold dilutions from schistosomes' DNA were prepared with ultrapure water ranging from 1 × 10^−1^ to 1 × 10^−9^ and stored at -20°C until use. DNAs thus prepared were used as positive controls in all LAMP and PCR reactions as well as for assessing sensitivity and specificity of both assays.

### 2.4. PCR with F3 and B3 External Primers

A touchdown PCR (TD-PCR) using designated F3 and B3 external primers was initially tested to verify that the correct target sequence selected *in silico* was amplified. The PCR assay was conducted in a 25 *μ*L reaction mixture containing 2.5 *μ*L of 10x buffer, 1.5 *μ*L of 25 mM MgCl_2_, 2.5 *μ*L of 2.5 mM dNTPs, 0.5 *μ*L of 100 pM F3 and B3, 2 U *Taq*-polymerase, and 2 *μ*L (10 ng) of DNA template. Initial denaturation was conducted at 94°C for 1 min, followed by a touchdown program for 15 cycles with successive annealing temperature decrements of 1.0°C every 2 cycles. For these 2 cycles, the reaction was denatured at 94°C for 20 s followed by annealing at 65°C–60°C for 20 s and extension at 72°C for 30 s. The following 15 amplification cycles were similar, except that the annealing temperature was 59°C. The final extension was performed at 72°C for 10 min. The same reaction mixture was used in all PCR reactions (except for the primers), and the amplification conditions varied according to different annealing temperatures of the primers used.

DNA samples (2 *μ*L; 0.5 ng/*μ*L) from the *Schistosoma* species included were used to evaluate specificity; negative (ultrapure water instead of DNA) and positive (DNA from each species) controls were included in each PCR assay.

### 2.5. LAMP Assay

The LAMP primer sets designed were evaluated by using a reaction mixture containing 40 pmol each of FIP and BIP primers, 5 pmol each of F3 and B3 primers, 1.4 mM each of dNTP (Intron), 1x Isothermal Amplification Buffer-20 mM Tris-HCl (pH 8.8), 50 mM KCl, 10 mM (NH_4_)_2_SO_4_, 2 mM MgSO_4_, 0.1% Tween20 (New England Biolabs, UK)-betaine (1 M) (Sigma, USA), supplementary MgSO_4_ (4 mM) (New England Biolabs, UK), and 8 U of *Bst* polymerase 2.0 WarmStart (New England Biolabs, UK) with 2 *μ*L (1 ng) of template DNA. LAMP reactions were performed in 0.2 mL tubes that were incubated in a dry bath heat block at 63°C-65°C for 60 min and then heated at 80°C for 5-10 min to stop the reaction.

Schistosome DNA samples mentioned above were used to evaluate the specificity of the LAMP assay; the lower detection limit of the LAMP assay was established by using 10-fold serial dilutions prepared as previously described. Positive controls (DNA from all species tested) and negative controls (ultrapure water instead of DNA) were included in all LAMP reactions.

### 2.6. Detection of Amplification Products

PCR amplification products were monitored using 1.5% agarose gel electrophoresis stained with ethidium bromide and visualised under UV light.

LAMP reaction results were visually inspected by colorimetric change by adding 2 *μ*L (1 : 10, 10,000x) SYBR Green I fluorescent dye (Invitrogen, Carlsbad, California, USA) to the reaction tubes. Green fluorescence was observed in positive reactions whilst it remained original orange in negative reactions; additionally, the products (3-5 *μ*L) were monitored by 1.5% agarose gel electrophoresis and visualised under UV light. All electrophoresed PCR and LAMP agarose gels were photographed using an ultraviolet gel documentation system (UVItec, UK).

## 3. Results

### 3.1. Selecting Targets for LAMP Amplification of *Schistosoma bovis* and Genus *Schistosoma*

**Figure 1:**
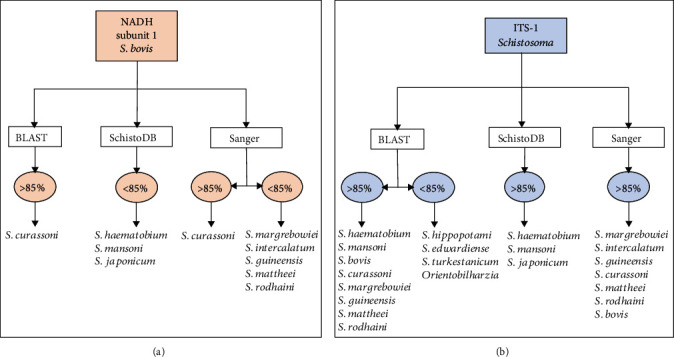
Degree of sequence similarity detected amongst the selected sequences for designing the LAMP primers and schistosome sequences queried in each database. (a) *S. bovis* mitochondrial NADH subunit 1 sequence. (b) ITS-1 sequence from several *Schistosoma* species.

Sequence similarity analysis of the selected sequences downloaded from the GenBank, SchistoDB, and Sanger databases allowed selecting several potentially useful sequences to design primers for the specific detection by LAMP of *S. bovis* and for the simultaneous detection of several schistosome species (genus *Schistosoma*) (Tables S1, S2). After comparison, a 678 bp sequence derived from mitochondrial NADH subunit 1 (NADH-1) (GenBank access number HM594942) and a 457 bp sequence from the internal transcribed spacer 1 (ITS-1) (GenBank access number GU257398) were selected for detecting *S. bovis* and *Schistosoma* spp., respectively (Figure 1).

### 3.2. Designing and Synthesising Primers for LAMP Amplification

Specific LAMP primers were designed using two programs: LAMP Designer and PrimerExplorer V5. Different primer sets were generated depending on the particular characteristics and parameters evaluated by each software. A set of 6 primers (including 2 loop primers) were thus selected to amplify the *S. bovis* NADH-1 sequence as designed by the LAMP Designer software, whilst a set of 5 primers (including 1 loop primer) to amplify *Schistosoma* species ITS-1 sequence, as designed in the PrimerExplorer software (Table 1).

### 3.3. TD-PCR with F3 and B3 External Primers

**Figure 2:**
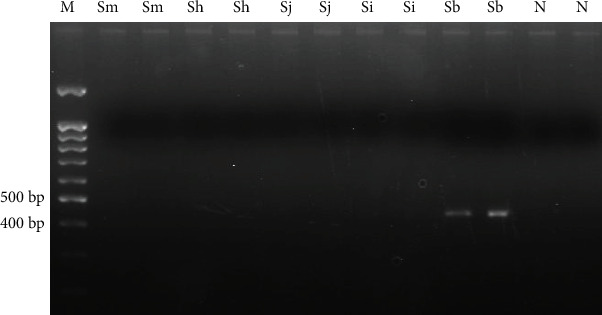
TD-PCR F3-B3 for amplifying *S. bovis* NADH-1. A 58-53°C temperature range and 53°C × 15 cycles were used. Sm: *S. mansoni* DNA; Sh: *S. haematobium* DNA; Sj: *S. japonicum* DNA; Si: *S. intercalatum* DNA; Sb: *S. bovis* DNA; N: negative control (ultrapure water, no DNA). M: molecular weight marker (100 bp PLUS BLUE DNA ladder).

After testing several different reaction temperatures and cycles, amplification conditions for TD-PCR were finally established for *S. bovis* NADH-1 (range 58-53°C; 53°C × 15 cycles) and *Schistosoma* spp. ITS-1 fragment (range 61-57°C; 57°C × 30 cycles). An approximately 420 bp PCR product was obtained for *S. bovis* NADH-1 sequence (400 bp predicted *in silico*) (Figure 2). This sequence was only amplified when using *S. bovis* DNA, but no amplicons were obtained with DNA samples from other schistosome species tested.

**Figure 3:**
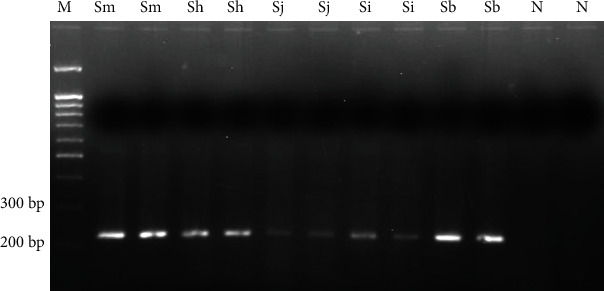
TD-PCR F3-B3 for amplifying genus *Schistosoma* ITS-1. A 61-57°C temperature range, and 57°C × 30 cycles were used. Sm: *S. mansoni* DNA; Sh: *S. haematobium* DNA; Sj: *S. japonicum* DNA; Si: *S. intercalatum* DNA; Sb: *S. bovis* DNA; N: negative control (ultrapure water, no DNA). M: molecular weight marker (100 bp PLUS BLUE DNA ladder).

On the other hand, a PCR product between 220 and 225 bp was obtained for *Schistosoma* spp. ITS-1 sequence (216 bp predicted *in silico*) (Figure 3). This PCR product was successfully amplified when DNA samples from the schistosome species included in the study were analysed. However, amplicons obtained for *S. mansoni*, *S. haematobium*, and *S. bovis* showed a greater signal intensity than those obtained for *S. japonicum* and *S. intercalatum.*

### 3.4. LAMP for Amplifying *S. bovis* NADH-1 and Genus *Schistosoma* ITS-1 Target Sequences

**Figure 4:**
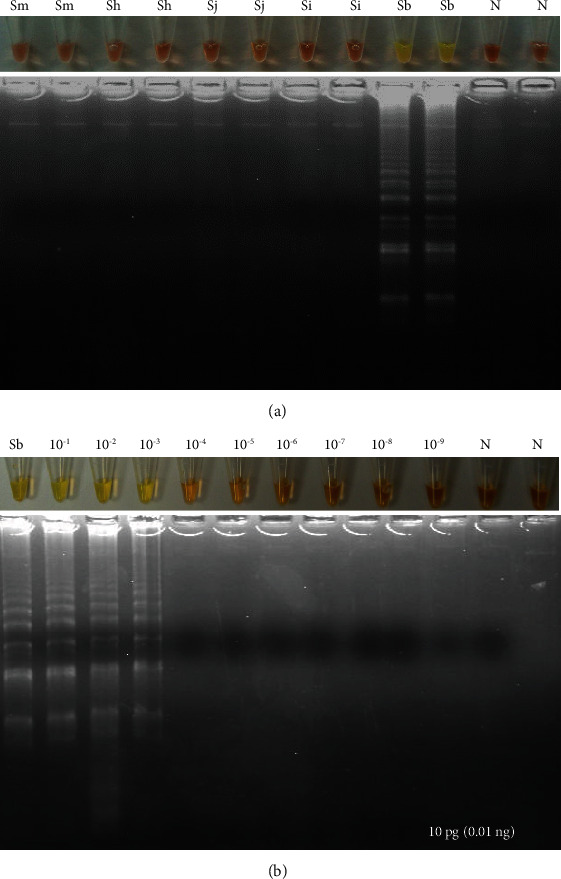
LAMP assay for amplifying *S. bovis* NADH-1. (a). Specificity assessment. Only NADH-1 was amplified using *S. bovis* DNA. Sm: *S. mansoni* DNA; Sh: *S. haematobium* DNA; Sj: *S. japonicum* DNA; Si: *S. intercalatum* DNA; Sb: *S. bovis* DNA; N: negative controls (ultrapure water, no DNA). (b). Sensitivity assessment. Sb: *S. bovis genomic* DNA (10 ng/*μ*L); lanes 10^−1^-10^−9^, 10-fold serially dilutions.

As shown in Figure 4, only LAMP products were obtained when *S. bovis* DNA was used as template to amplify NADH-1 sequence. No false positive amplification was observed when using DNA from other schistosomes (Figure 4(a)), thus indicating the high specificity of the designed LAMP primers. Regarding sensitivity, the results indicated that the detection limit of LAMP for *S. bovis* NADH-1 amplification was 0.01 ng (10 pg) (Figure 4(b)).

**Figure 5:**
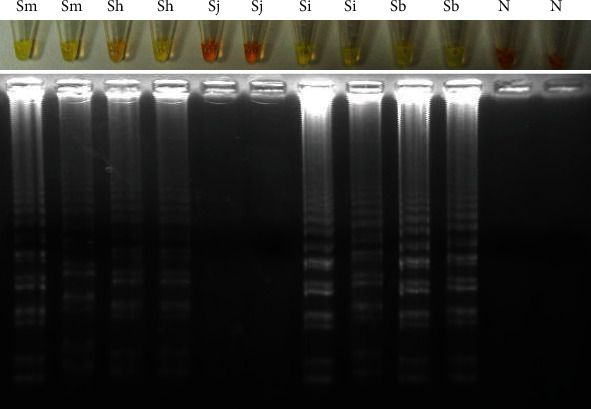
LAMP for amplifying the genus *Schistosoma* ITS-1 sequence. Lanes Sm, Sh, Sj, Si, and Sb mean *S. mansoni*, *S. haematobium*, *S. japonicum*, *S. intercalatum*, and *S. bovis* DNAs, respectively; Lanes N: negative controls (ultrapure water, no DNA).

LAMP results when using the specific primers to amplify ITS-1 sequence for several schistosome species DNA are shown in Figure 5. Amplification products were observed when using DNA from *S. mansoni*, *S. haematobium*, *S. intercalatum*, and *S. bovis*, but not from *S. japonicum*. Colour change was clearly visualised in positive results, and also, a typical ladder-like band pattern was observed on agarose gel electrophoresis.

**Figure 6:**
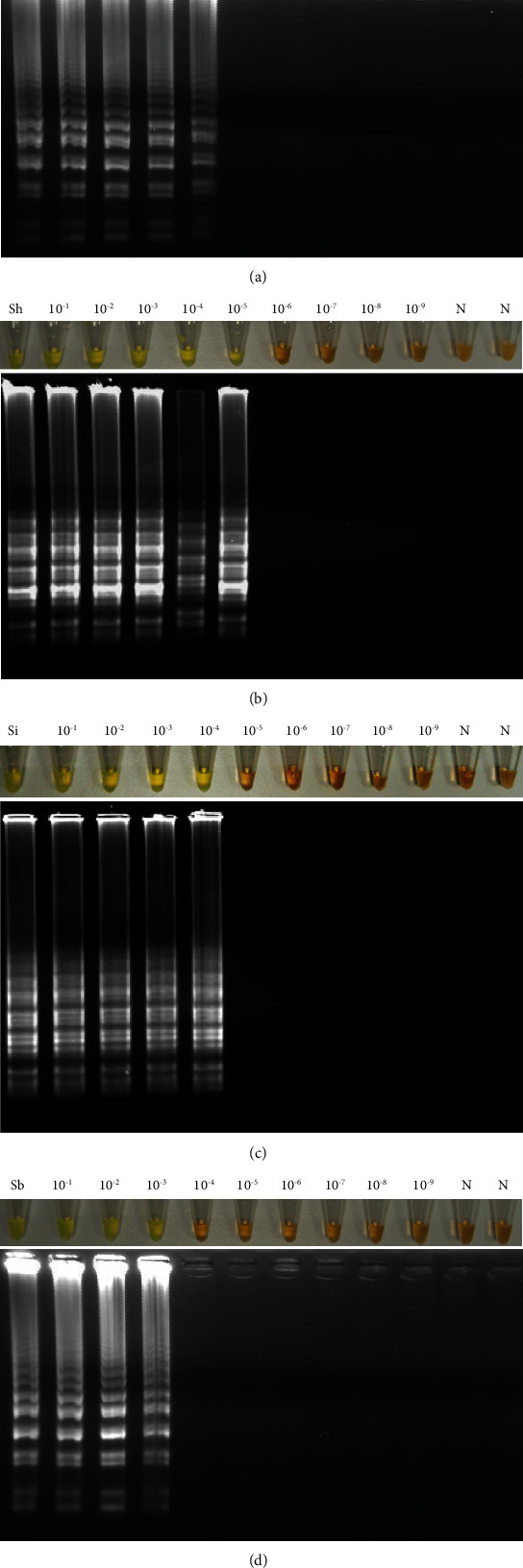
Sensitivity assessment of LAMP for amplifying *Schistosoma* ITS-1 sequence using DNA from different schistosome species. (a) Detection limit for *S. mansoni* (1 pg). (b) Detection limit for *S. haematobium* (0.1 pg). (c) Detection limit for *S. intercalatum* (1 pg). (d) Detection limit for *S. bovis* (10 pg). Lanes Sm, Sh, Si, and Sb mean *S. mansoni*, *S. haematobium*, *S. intercalatum*, *and S. bovis* DNAs, respectively. Lanes 10^−1^-10^−9^: 10-fold serial dilutions of DNA. Lanes N: negative controls (no DNA template).

When evaluating the sensitivity of the established LAMP assays for ITS-1 sequence, the detection limit in *Schistosoma* spp. genomic DNA amplification was different depending on the species used as template (Figure 6). Thus, a detection limit of 0.001 ng (1 pg) was obtained for *S. mansoni* and *S. intercalatum* (Figures 6(a) and 6(c)), 0.0001 ng (0.1 pg) for *S. haematobium* (Figure 6(b)), and 0.01 ng (10 pg) for *S. bovis* (Figure 6(d)).

## 4. Discussion

Schistosomiasis is a neglected tropical disease widespread in 74 tropical and subtropical countries. The most important schistosome species regarding human schistosomiasis are *S. japonicum* in the Republic of China, the Philippines, and Indonesia; *S. haematobium* in Africa and in some countries of the Arabian peninsula (it has also recently emerged on the French island of Corsica); and *S. mansoni* in Africa, the Arabian peninsula, and Latin America. Meanwhile, *S. guineensis* and *S. intercalatum* (both endemic in Central and West Africa) and *S. mekongi* (restricted to a short stretch of the Mekong River in southern Lao People's Democratic Republic and eastern Cambodia) are of local regional importance [21]. The disease can also cause chronic, debilitating infection in animals, and it has been estimated that more than 165 million cattle are infected worldwide causing high levels of morbidity amongst susceptible animals (cattle, goats, sheep, horses, camelids, and pigs) and causing considerable production losses due to liver damage, reduced reproductive performance/yields, increased susceptibility to other infectious agents, and death [9]. There is no data on the current prevalence of *S. bovis* (animal schistosomiasis), but in the past, it had a wide distribution and prevalence in many Mediterranean, African, and Asian countries [22].

Hybridisations between schistosomes have already been identified between different human-specific schistosome species, different animal-specific schistosome species, and between human-specific and animal-specific schistosome species. The hybrid forms between human-specific and animal-specific schistosome species are particularly startling because they raise the possibility of the spread of hybrids, particularly zoonotic hybrids, that could prove problematic in terms of maintaining transmission if the can replace existing species and parasite strains, extend intermediate and definitive host ranges, or present and increase infectivity and virulence [23].

In addition, the hybrid status of the parasite may impair the parasitological, serological, and molecular diagnostic.

This work provides new LAMP assays for the specific detection of *S. bovis* and, additionally, for the simultaneous detection of a number of other human-infecting *Schistosoma* species.

The LAMP method designed for the simultaneous detection of different species of the *Schistosoma* genus achieved DNA amplification of four of the five species including *S. mansoni*, *S. haematobium*, *S. intercalatum*, and *S. bovis*; all of which are found in Africa. The *S. japonicum* DNA could not be amplified, possibly due to the few—although determinant—differences that its sequence presents with respect to DNA sequences from other African schistosomes, which share higher levels of identity [24, 25]. However, when performing the TD-PCR with the external primers F3 and B3 to check the *in silico* size of the selected ITS-1 sequence, it was possible to amplify the DNA of all species analysed, including *S. japonicum*. This could be explained by the additional primers required for the amplification in the LAMP technique, so internal primers might have not annealed in the *S. japonicum* DNA sequence. Moreover, it is important to highlight that due to the different origin of African and Asian schistosomes, it is very difficult to design primers for the amplification of common sequences amongst all species taking into account geographical variation [26, 27]. On the other hand, the ITS-1 sequence type selected for primer design was that obtained from *S. haematobium* and, for that reason, a higher degree of identity is expected for all African species, whilst Asian species share less identity.

Regarding the sensitivity of the developed LAMP methods, the detection limit of the LAMP for the *S. bovis* NADH-1 amplification was 10 pg of genomic DNA. On the other hand, the detection limit achieved with the LAMP for the amplification of the ITS-1 region of the *Schistosoma* genus was found to be 1 pg, 0.1 pg, 1 pg, and 10 pg for *S. mansoni*, *S. haematobium*, *S. intercalatum*, and *S. bovis*, respectively. These differences in the sensitivity obtained for the different species may be due to the small differences between the nucleotide sequences that may end up influencing primer annealing and, therefore, LAMP performance. It makes sense that *S. haematobium* is the species displaying the highest detection limit (0.1 pg), since its own sequence was used as a reference when designing the generic LAMP.

In terms of specificity, the LAMP designed for *S. bovis* NADH-1 amplification worked specifically for this species and no DNA from other schistosome species included in the study was amplified. On the other hand, the *Schistosoma* genus ITS-1 region LAMP amplified the DNA of all species tested except for *S. japonicum.* A LAMP method for the specific detection of *S. bovis* and another for the simultaneous detection of different schistosome species that can produce human schistosomiasis have thus been developed. It should be noted that comparisons of genetic distances from pairs of congeners for both mitochondrial DNA and ITS sequences have demonstrated that mitochondrial DNA sequences of platyhelminths (including schistosomes) accumulate nucleotide substitutions at a much higher rate than ITS [28]. Moreover, until now, most of the *S. haematobium-S. bovis* hybrids reported demonstrate the existence of a mitochondrial DNA (i.e., cox 1 and microsatellite DNA) introgressive hybridisation of *S. haematobium* by *S. bovis* [29]. Thus, our developed LAMP assay based on NADH-1 for *S. bovis* detection would be very useful for detecting *S. haematobium-S. bovis* hybrids, as most hybrids have a mitochondrial *S. bovis* signature and a *S. haematobium* ITS signature.

## 5. Conclusions

Two highly sensitive LAMP methods for detecting different *Schistosoma* species important for human and veterinary health were standardised. It is worth highlighting that LAMP assays are easier to turn into point-of-care tests since no specialised lab equipment is required. Considering that human cases due to *S. intercalatum* are currently increasing and hybridisation events between *S. bovis* and *S. haematobium* have been reported in Senegal and France [22], LAMP methods here developed could be very useful for the diagnosis and surveillance of schistosome infections.

## Figures and Tables

**Table 1a:** (a) LAMP primers for the *S. bovis* sequence (LAMP Designer)

NADH subunit 1						
Primer	5′ pos	3′ pos	Length (bp)	Tm (°C)	GC ratio (%)	Sequence
SbF3	219	236	18	56.8	38.9	TTCATTGTTAGGTTGCGT
SbB3	642	619	24	57	33.3	TCTATATTCTACTCTAATCCCTCT
SbFIP			48			TCAGTATCATCTCAAACATCACACTAGTAGTATGTTCTGTCTTAAGTT
SbBIP			45			TTTGTAGTACCTCTGGTTTACATCATTCACTCTCAGACTCTACAT
SbF2	327	349	23	56.8	30.4	AGTAGTATGTTCTGTCTTAAGTT
SbF1c	424	448	25	62.1	36	TCAGTATCATCTCAAACATCACACT
SbB2	593	612	20	57	40	TTCACTCTCAGACTCTACAT
SbB1c	520	544	25	61.8	36	TTTGTAGTACCTCTGGTTTACATCA
SbLF	364	388	25	61.9	36	ACTTAGACCATGAACATCAACCTAT
SbLB	560	584	25	61.9	40	TACTAAGTGAGAGTAATCGAACACC

**Table 1b:** (b) LAMP primers for the genus *Schistosoma* sequence (Primer Explorer V5)

ITS-1						
Primer	5′ pos	3′ pos	Length (bp)	Tm (°C)	GC ratio (%)	Sequence
SF3	2	19	18	59.7	61	TTGACCGGGGTACCTAGC
SB3	200	218	19	59.5	53	CGTGAATGGCAAGCCAAAC
SFIP			39			ATCGCCCTTGGCAGATCAGGCTGTCGTATGCCCTGATGG
SBIP			40			ATATGCATGCAAATCCGCCCCGCGGATCGCTTCAACAGTGTA
SF2	20	38	19	59.2	58	CTGTCGTATGCCCTGATGG
SF1c	61	80	20	64.2	60	ATCGCCCTTGGCAGATCAGG
SB2	180	199	20	59.5	50	CGGATCGCTTCAACAGTGTA
SB1c	135	156	20	65.9	55	ATATGCATGCAAATCCGCCCCG
SLF	39	60	22	60.4	45	CAGATCAGGCAACCCGAAAG

For *S. bovis* (Sb) and genus *Schistosoma* (S): F3=forward outer primer; B3=backward outer primer; FIP=forward inner primer (comprising F1c and F2 sequences); BIP=backward inner primer (comprising B1c and B2 sequences); LF=loop forward primer; LB=loop backward primer.

## Data Availability

The data used to support the findings of this study are included within the article.

## Supplementary Materials

Supplementary Materials Table 1: gene sequences located and analysed for *S. bovis.* Table 2: degree of ITS-1 and ITS-2 sequence identity regarding schistosome species in the different databases.
