# Supplementary material for: Molecular Markers for Detecting Schistosoma Species by Loop-Mediated Isothermal Amplification
Source: Dis Markers. 2020 Jul 22;2020:8042705. doi: 10.1155/2020/8042705 (PMC7396120; doi:10.1155/2020/8042705)
Supplement: Supplementary Materials — Table 1: gene sequences located and analysed for S. bovis. Table 2: degree of ITS-1 and ITS-2 sequence identity regarding schistosome species in the different databases. [file 8042705.f1.pdf]

Supplementary material

Table 1. Gene sequences located and analysed for *S. bovis*

***S. bovis* sequences**	**Degree of identity according to databases**		
	**BLAST at NCBI**	**SchistoDB**	**Sanger**
rRNA5-8S<br>genome (155 bp)<br>FJ588862	Genus *Schistosoma*	100% *S. mansoni*<br>99% *S. haematobium*<br>100% coverage	96% *S. curassoni*<br>100% coverage
18S rRNA<br>genome (1,864 bp)<br>AY157238	Genus *Schistosoma*	99% *S. mansoni*<br>93% *S. haematobium*<br>100% coverage	99% *S. curassoni*<br>100% coverage
rRNA-28S<br>genome (3,829 bp)<br>AY157266	Genus *Schistosoma*	98% *S. mansoni*<br>86% *S. haematobium*<br>100% coverage	99% *S. curassoni*<br>100% coverage
ITS 1<br>genome, between 18S and 5.8S (457 bp) FJ588862	Genus *Schistosoma*	95% *S. mansoni*<br>87% *S. haematobium*<br>100% coverage	98% *S. curassoni*<br>100% coverage
ITS 2<br>genome, between 5.8S and 28S (313 bp)<br>AF146030	Genus *Schistosoma*	93% *S. mansoni*<br>and *S. haematobium*<br>100% coverage	97% *S. curassoni*<br>100% coverage
Tetraspanin 23<br>genome<br>(1,620 bp)<br>JX844194	Genus *Schistosoma*	94% *S. haematobium*<br>85% *S. mansoni*<br>100% coverage	89% *S. curassoni*<br>91% *S. mattheei*<br>85% *S. intercalatum*<br>100% coverage
Tetraspanin 23<br>genome<br>(1,617 bp)<br>JX844196	Genus *Schistosoma*	93% *S. haematobium*<br>86% *S. mansoni*<br>100% coverage	89-99% *S. curassoni*<br>92-95 % *S. mattheei*<br>83-95% *S. intercalatum*<br>100% coverage
Tetraspanin 23<br>genome<br>(291 bp<br>- 1,620 bp)<br>JX844194	No homology with other schistosomes	89% *S. haematobium*<br>87% *S. mansoni*<br>87% *S. japonicum*<br>Overlapping in small parts of the sequence	60-61% *S. intercalatum*<br>60-65% *S. margrebowiei*<br>61-65% *S. curassoni*<br>60% *S. guineensis*<br>65% *S. mattheei*<br>57-60% *S. rodhaini*<br>100% coverage
14-3-3 protein<br>genome<br>(832 bp)<br>DQ176433	Genus *Schistosoma*	99% *S. haematobium*<br>92% *S. mansoni*<br>100% coverage	98% *S. curassoni*<br>100% coverage
Glutathione S-transferase genome<br>(781 bp)<br>M87800	Genus *Schistosoma*	99% *S. haematobium*<br>90% *S. mansoni*<br>100% coverage	98% *S. curassoni*<br>100% coverage

Repeat sequence 1 genome (203 bp) DQ831697	*S. curassoni*, *S. mattheei* and *S. haematobium*	85%–95% *S. haematobium*<br>76%–83% *S. mansoni*<br>No 100% coverage	82-100% *S. curassoni*<br>80% *S. mattheei*<br>100% coverage
Repeat sequence 2 genome (227 bp) DQ831700	*Schistosoma haematobium* group	94% *S. haematobium*<br>100% coverage	85-95% *S. curassoni*<br>87-94% *S. mattheei*<br>92-93% *S. margrebowiei*<br>100% coverage
Repeat sequence 3 genome (161 bp) DQ831699	Genus *Schistosoma*	94% *S. haematobium*<br>89% *S. mansoni*<br>100% coverage	89% *S. curassoni*<br>92% *S. margrebowiei*<br>100% coverage
Repeat sequence 4 genome (216 bp) Mitochondrial	*Schistosoma haematobium* group	95% *S. haematobium*<br>Almost100% coverage	94-96% *S. curassoni*<br>95-100% *S. mattheei*<br>85-97% *S. margrebowiei*<br>100% coverage
COX-1 DQ831698 (503 bp) AY318827	Genus *Schistosoma*	90% *S. haematobium*<br>80% *S. mansoni*<br>100% coverage	65-77% *S. curassoni*<br>83% *S. guineensis*<br>55-75% *S. mattheei*<br>100% coverage
Mitochondrial COX-1 (1,224 bp) AJ519521	Genus *Schistosoma*	89% *S. haematobium*<br>80% *S. mansoni*<br>100% coverage	72-94% *S. curassoni*<br>84% *S. guineensis*<br>73% *S. mattheei*<br>100% coverage
Mitochondrial 12S rRNA (801 bp) FJ897168	Genus *Schistosoma*	84-95% *S. haematobium*<br>84% *S. mansoni*<br>100% coverage	98% *S. curassoni*<br>91-94% *S. guineensis*<br>100% coverage
Mitochondrial 16S rRNA (390 bp) EU513556	Genus *Schistosoma*	93% *S. haematobium*<br>84% *S. mansoni*<br>100% coverage	96% *S. curassoni*<br>100% coverage
	**NADH**		
Mitochondrial NADH subunit 6 (592 bp) FJ897164	*S. curassoni* and *S. haematobium* (100% and 94% coverage and 80% identity)<br>*S. guineensis* (92% identity and 70% coverage)	84% *S. haematobium*<br>69 -86% *S. mansoni*<br>100% coverage<br>81%-93% *S. japonicum* (overlapping only occurred in very small regions)	85% *S. curassoni*<br>62-67% *S. margrebowiei*<br>70% *S. intercalatum*<br>65-75% *S. guineensis*<br>62-65% *S. mattheei*<br>100% coverage
Mitochondrial NADH consensus x3 (2,215 bp) Sub 4, 3, 1	*Schistosoma haematobium* group	81% *S. haematobium*<br>70% *S. mansoni*<br>Almost100% coverage<br>68% *S. japonicum*<br>No 100% coverage	92% *S. curassoni*<br>56-68% *S. margrebowiei*<br>56-75% *S. guineensis*<br>56-68% *S. mattheei*<br>100% coverage

Mitochondrial NADH subunit 4 (700 bp) HM594942	*S. curassoni* (100%), *S. margrebowiei* (75%) and 95% coverage	82% *S. haematobium*<br>68 -83% *S. mansoni*<br>100% coverage<br>76%-86% *S. japonicum*, (overlapping only occurred in very small regions)	88-92% *S. curassoni*<br>75% *S. margrebowiei*<br>78% *S. intercalatum*<br>75% *S. guineensis*<br>59-68% *S. mattheei*<br>100% coverage
Mitochondrial NADH subunit 3 (369 bp) HM594942	*S. curassoni* (94% identity and 100% coverage)	76% *S. mansoni*<br>75% *S. japonicum*<br>136-362 bp overlapping<br>88%-95% *S. haematobium* and almost 100% coverage	95% *S. curassoni*<br>70-85% *S. margrebowiei*<br>75% *S. intercalatum*<br>73% *S. guineensis*<br>79% *S. mattheei*<br>100% coverage
Mitochondrial NADH subunit 1 (678 bp) HM594942	*S. curassoni* (94% identity and 99% overlapping)	70% *S. mansoni*<br>66% *S. japonicum*<br>83% *S. haematobium*<br>Almost 100% coverage in all	92% *S. curassoni*<br>58-68% *S. margrebowiei*<br>78% *S. intercalatum*<br>72% *S. guineensis*<br>78% *S. mattheei*<br>70% *S. rodhaini*<br>100% coverage

*Specific sequences selected for each case are highlighted in colour.

Table 2. Degree of ITS-1 and ITS-2 sequence identity regarding schistosome species in the different databases

***Schistosoma* spp. sequence**	**Degree of identity according to BLAST databases at NCBI**	**SchistoDB**	**Sanger**
ITS-2 nuclear genome, between 5.8S and 28S (311 bp) GU257398	All species from the genus *Schistosoma*	92% *S. mansoni*<br>91% *S. haematobium*<br>81% *S. japonicum*<br>100% coverage	89-97% *S. bovis*<br>90-92% *S. intercalatum*<br>91% *S. curassoni*<br>89-93% *S. guineensis*<br>90-95% *S. margrebowiei*<br>78-96% *S. mattheei*<br>81-93% *S. rodhaini*<br>100% coverage
ITS-1 nuclear genome, between 18S and 5.8S (457 bp) GU257398	All species from the genus *Schistosoma*	95% *S. mansoni*<br>88% *S. haematobium*<br>86% *S. japonicum*<br>100% coverage	84-98 % *S. bovis*<br>81-99% *S. intercalatum*<br>77-98% *S. curassoni*<br>81-96% *S. guineensis*<br>96-98% *S. margrebowiei*<br>78- 97% *S. mattheei*<br>85-96% *S. rodhaini*<br>100% coverage

* Specific sequences selected for each case are highlighted in colour
